# A Teratocarcinoma-Like Human Embryonic Stem Cell (hESC) Line and Four hESC Lines Reveal Potentially Oncogenic Genomic Changes

**DOI:** 10.1371/journal.pone.0010263

**Published:** 2010-04-23

**Authors:** Outi Hovatta, Marisa Jaconi, Virpi Töhönen, Frédérique Béna, Stefania Gimelli, Alexis Bosman, Frida Holm, Stefan Wyder, Evgeny M. Zdobnov, Olivier Irion, Peter W. Andrews, Stylianos E. Antonarakis, Marco Zucchelli, Juha Kere, Anis Feki

**Affiliations:** 1 Division of Obstetrics and Gynecology, Department of Clinical Science, Intervention and Technology, Karolinska Institutet, Stockholm, Sweden; 2 Department of Pathology and Immunology, University of Geneva Medical School, Geneva, Switzerland; 3 Department of Biosciences, Karolinska Institutet, Stockholm, Sweden; 4 Department of Genetic Medicine and Development, University of Geneva Medical School and Geneva University Hospitals, Geneva, Switzerland; 5 Swiss Institute of Bioinformatics, Geneva, Switzerland; 6 Department of Obstetrics and Gynecology, Geneva University Hospitals, Geneva, Switzerland; 7 Centre for Stem Cell Biology and Department of Biomedical Science, University of Sheffield, Sheffield, United Kingdom; University of California Riverside, United States of America

## Abstract

The first Swiss human embryonic stem cell (hESC) line, CH-ES1, has shown features of a malignant cell line. It originated from the only single blastomere that survived cryopreservation of an embryo, and it more closely resembles teratocarcinoma lines than other hESC lines with respect to its abnormal karyotype and its formation of invasive tumors when injected into SCID mice. The aim of this study was to characterize the molecular basis of the oncogenicity of CH-ES1 cells, we looked for abnormal chromosomal copy number (by array Comparative Genomic Hybridization, aCGH) and single nucleotide polymorphisms (SNPs). To see how unique these changes were, we compared these results to data collected from the 2102Ep teratocarcinoma line and four hESC lines (H1, HS293, HS401 and SIVF-02) which displayed normal G-banding result. We identified genomic gains and losses in CH-ES1, including gains in areas containing several oncogenes. These features are similar to those observed in teratocarcinomas, and this explains the high malignancy. The CH-ES1 line was trisomic for chromosomes 1, 9, 12, 17, 19, 20 and X. Also the karyotypically (based on G-banding) normal hESC lines were also found to have several genomic changes that involved genes with known roles in cancer. The largest changes were found in the H1 line at passage number 56, when large 5 Mb duplications in chromosomes 1q32.2 and 22q12.2 were detected, but the losses and gains were seen already at passage 22. These changes found in the other lines highlight the importance of assessing the acquisition of genetic changes by hESCs before their use in regenerative medicine applications. They also point to the possibility that the acquisition of genetic changes by ESCs in culture may be used to explore certain aspects of the mechanisms regulating oncogenesis.

## Introduction

Human embryonic stem cells (hESCs) and human embryonal carcinoma cells (hECs) are two pluripotent cell types that share many characteristics [1] Human ECs are the malignant stem cells of teratocarcinomas, which are malignant tumors that have embryonal carcinoma components, and some may form teratocarcinomas when re-transplanted into an animal [2]. Both hECs and hESCs can differentiate into many cell types, but the differentiation potential of hECs is limited compared to that of hESC lines [1], [3], [4]. Before the clearly malignant line CH-ES1 [5] was developed, human ESC lines were reported to be benign; they form teratomas comprising differentiated tissue components of the three embryonic germ layers after injection into immune-incompetent mice, but they usually do not form teratocarcinomas. After culture adaptation, hESC lines can develop malignant features [6], but their ability to form tumors has not been analyzed in detail.

Human ESC lines have been most often derived from the inner cell mass of blastocyst-stage embryos [7] but they have also been derived from eight-cell stage morula embryos [8]. Klimanskaya et al. derived hESC lines from single isolated blastomeres at first by co-culture with other hESCs [9] but they were subsequently able to do so without such support [10]. These lines had normal karyotypes, and they formed teratomas when grown as xenografts. In another study, Van de Velde et al. [11] were able to obtain pluripotent cell lines from single blastomeres derived from four-cell stage embryos. These embryos had been established for this purpose and were of good quality. Nonetheless, the first line was karyotypically abnormal.

We established an hESC line from the only surviving blastomere of a four-cell stage embryo. This single cell survived freezing and thawing [5] and produced a cell line expressing the typical markers of hECs and hESCs. This line proved to be chromosomally very abnormal and was highly invasive when transplanted into SCID mice [5]. Hence, it has characteristics more similar to hECs than to hESCs.

In the present study, we have characterized the genomic changes that may explain the enhanced oncogenicity of the CH-ES1 teratocarcinoma-like hESC line relative to other pluripotent cell lines. We used both comparative genomic hybridization (aCGH) and single nucleotide polymorphism (SNP) genotyping to detect genomic changes in the CH-ES1 line, the 2102Ep teratocarcinoma line and four benign hESC lines (H1, HS293, HS401 and SIVF-02) originating from three different laboratories. In addition to finding extensive genomic abnormalities in the CH-ES1 line, we also found that the H1, HS293, HS401 and SIVF-02 lines share the general characteristics of hESCs that have been described by the International Stem Cell Initiative (ISCI) 1 [12]. We observed suggestive culture adaptation and growth advantages in these lines, as well as gains of known oncogenes and the possible deletion or loss of putative yet unrecognized tumor suppressor genes. The SNP arrays also revealed potentially tumorigenic changes in the karyotypically normal hESC lines.

## Results

**Figure 1:**
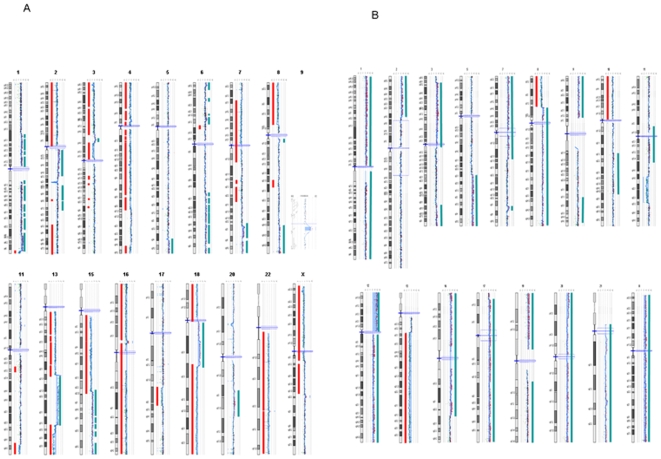
Cartography of genetic aberrations which were found in the CH-ES1 and, 2102Ep cell lines. A) Line CH-ES1, B) Line 2102Ep. Blue bars show duplicated regions and red ones show deleted regions.

In the teratocarcinoma line 2102Ep and in the teratocarcinoma-like line CH-ES1, the chromosomal complement was highly aneuploid (Figure 1). CGH analysis of CH-ES1 confirmed a high level of genomic imbalances in agreement with earlier G-banding results. We performed a high resolution CGH analysis that revealed a higher frequency of genomic losses compared to gains in CH-ES1 (Table 1). The regions of reduced copy number ranged from 6 to 88 Mb and involved chromosomes 1q, 3p, 4p, 4q, 8p, 11q, 13q, 15q, 16, 17q, and 18p. Duplicated regions (0.4 to 60 Mb) were seen in chromosomes 2q, 5q, 6p, 6q, 7q, 8q, 9q, 13q, 15q, and 18q (Table 2). There were partial trisomies of chromosomes 1, 9, 12, 19, 20 and X, and a duplication of 17p13.2-qtel (3.674-tel). Only chromosome 14 was normal in the CGH assay. When compared to the 2102Ep teratocarcinoma line in the CGH assay, CH-ES1 displayed two common large aberrations, namely, duplication in 5q34 and a deletion of 13q32.1-q34 (Table 2).

**Table 1:** Genetic aberrations found in the H1, CH-ES1, and 2102Ep cell lines.

Line	Number of genes	Status
2102 Ep	394	Deleted
	7665	Duplicated
CH-ES1	4019	Deleted
	1021	Duplicated
H1	71	Deleted
	1471	Duplicated

**Table 2:** Summary of the genomic aberrations detected in CH-ES1 and 2102Ep cell lines by CGH-Array.

2102 Ep cells			CH-ES1 cells		
Cytoband	Aberration	Size (Mb)	Cytoband	Aberration	Size (Mb)
1p36-p12	gain	124	1q44	loss	3.8
2p25-p16.	gain	52	2q11-q21.2	gain	39
3p26-p11	gain	73	3p26-p16.1	loss	60
5q23.1-q35	gain	62	3p14.1-p12.1	loss	17
7p22-q21.13	gain	88	4p16-q31.21	loss	79
8p23-p12	gain	30	4q34.3-q35	loss	9
9p24-p12	gain	51	5q34	gain	13
11q14.3-q23.3	loss	23	6p25	gain	4
12p13-q24.3	gain	132	6p22.3	gain	2
13q12.3-q34	loss	84	6p21.31-p21.2	gain	4
16p13.3-q23.2	gain	72	6q21-q27	gain	60
17q13.2-q25	gain	78	7q33-q36	gain	25
19p11.3-p12	gain	28	8p23-p12	loss	35
20p13-q13.3	gain	62	8q24.12 9q21.31	gain	25
21q22.11	gain	1.4	11q24.3	gain	0.4
Xp22.3-q28	gain	154	13q11q21.31	loss	6
			13q21-q32	loss	42
			13q32.1-q34	gain	33
			15q11q22.32	loss	10
			15q22.32-q26	loss	44
			16p13-q24	gain	37
			17q22q23.2	loss	88
			18p11	loss	9
			18q11-q12.3	loss	16
			18q12.3-q23	gain	20
				loss	39
					

**Figure 2:**
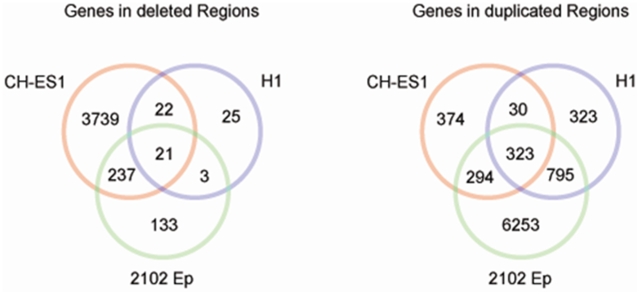
The number of genes in CH-ES1, 2102Ep and H1 lines in either deleted or duplicated regions. A) deleted regions, B) duplicated regions.

The CGH array showed extensive chromosomal changes in the teratocarcinoma line 2102Ep and in the malignant hESC line CH-ES1. There were also several visible changes in the H1 line at passage number 56, including partial 5 Mb duplications in 1q32.2 and 22q12.2, and these findings were confirmed by the SNP analysis. CGH analyses of the three other hESC lines did not identify any gains or deletions (Figure 2). A gene-level analysis revealed that, in CH-ES1, there were about 4019 hemizygously deleted genes and 1021 duplicated genes, whereas in 2102Ep, 394 genes were deleted and 7665 genes were duplicated. Similarly to other hESCs, control H1 cells showed about 71 deleted genes and 1471 duplicated genes (Table 1). When normal variations listed in the Genomic Variation databases [13] were excluded, 21 genes were deleted, and 323 were duplicated (Figure 2). The common deleted genes included BCL3, which is known to be mutated in B cell lymphomas (Table 3).

**Table 3:** Deleted genes in common between H1, CH-ES1, and 2102Ep cell lines.

Input ID	Entrez Gene ID	Symbol	Name
ALDH16A1	126133	ALDH16A1	aldehyde dehydrogenase 16 family, member A1
APOC1	341	APOC1	apolipoprotein C-I
APOC2	344	APOC2	apolipoprotein C-II
APOC4	346	APOC4	apolipoprotein C-IV
APOE	348	APOE	apolipoprotein E
BCAM	4059	BCAM	basal cell adhesion molecule (Lutheran blood group)
BCL3	602	BCL3	B-cell CLL/lymphoma 3
CBLC	23624	CBLC	Cas-Br-M (murine) ecotropic retroviral transforming sequence c
CCDC155			
CLPTM1	1209	CLPTM1	cleft lip and palate associated transmembrane protein 1
FCGRT	2217	FCGRT	Fc fragment of IgG, receptor, transporter, alpha
FLT3LG	2323	FLT3LG	fms-related tyrosine kinase 3 ligand
PIH1D1			
PTH2			
PVRL2	5819	PVRL2	poliovirus receptor-related 2 (herpesvirus entry mediator B)
RCN3	57333	RCN3	reticulocalbin 3, EF-hand calcium binding domain
RPL13A	23521	RPL13A	ribosomal protein L13a
RPS11	6205	RPS11	ribosomal protein S11
SLC17A7	57030	SLC17A7	solute carrier family 17 (sodium-dependent inorganic phosphate cotransporter), member 7
TOMM40	10452	TOMM40	translocase of outer mitochondrial membrane 40 homolog (yeast)

**Figure 3:**
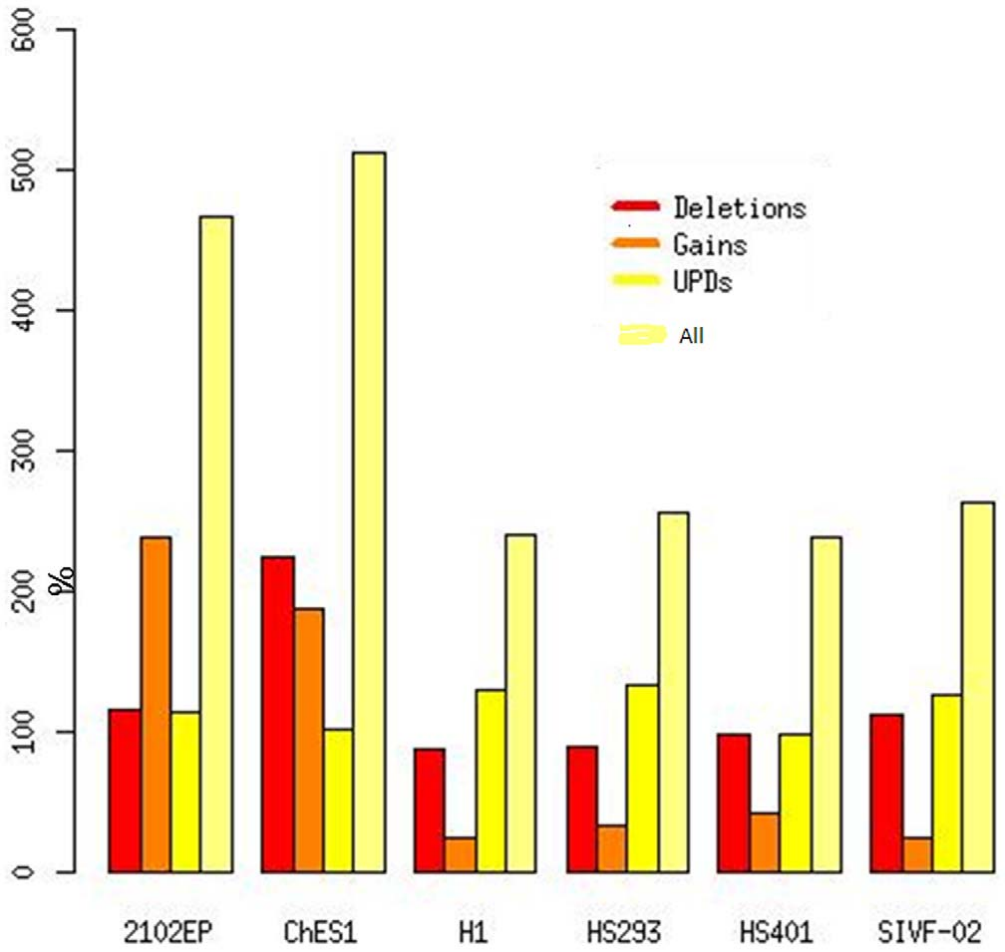
The number of aberrations per cell line detected by Affymetrix SNP6.0 arrays.

Further analyses using Affymetrix 6.0 SNP arrays confirmed all the changes observed by CGH and also identified an additional 1275 copy number variant sites. Of these, about 33% were shorter than 43 kb; the median resolution was 44 kb for the Agilent CGH arrays. About 60% of the CNVs were detected in the CH-ES1 and 2102Ep stem cell lines, consistent with their abnormal behavior (Figure 3).

**Figure 4:**
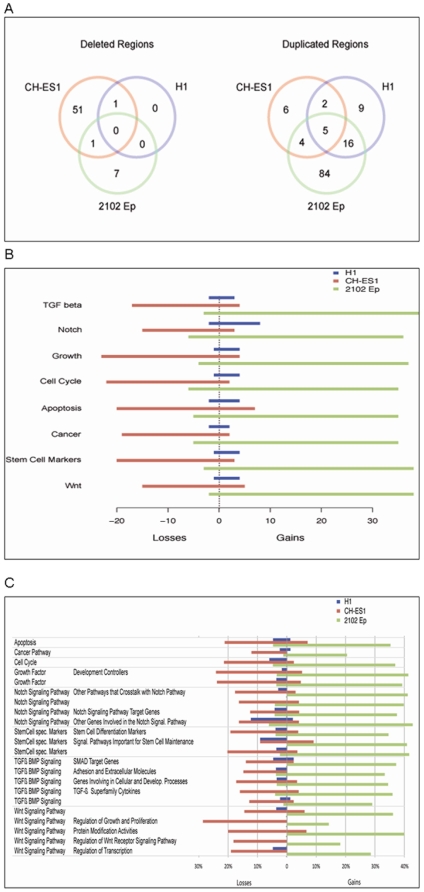
Pathway analyses of gained and lost genes in the analysed cell lines. A) The number of pathways which were statistically significantly enriched per line involving deleted (left panel) and duplicated (right panel) genes. B) The number of lost and gained genes per cell line in summary pathways. C) The percentage of genes altered per pathway by deletion or duplication. Only pathways with copy number variations are shown.

After assignment of the identified genes to KEGG pathways, we found that there were no common pathways among the 21 gene deletions shared by the H1, CH-ES1 and 2102Ep cells. However, there were five pathways that were altered among the 323 duplicated genes shared between these cell lines, including MAPK signaling, axon guidance, natural killer cell-mediated cytotoxicity, tight junction and Fc epsilon RI signaling pathways (Figure 4A). Almost all of these pathways were altered by gene deletions in CH-ES1 and gene duplications in 2102Ep (Figure 4B). However, we did not find any significantly predominant type of mutation in H1 (Figure 4C).

**Figure 5:**
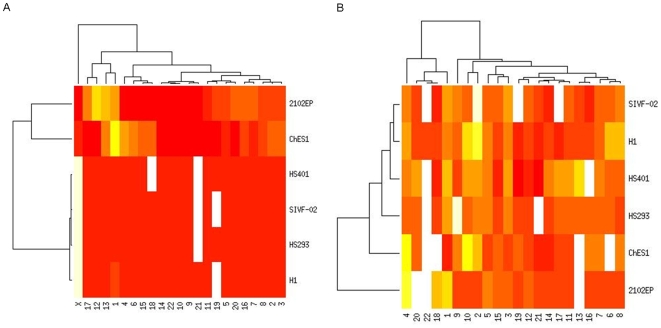
The number of CNVs and UPDs per chromosome and cell line as detected by Affymetrix 6.0 arrays. CH-ES1 and 2102Ep are the only female lines. A) The numer of CNVs, B) The number of UPDs.

Out of the 1275 CNVs detected by the SNP arrays, 165 were not previously reported in the database of Genomic Variants, suggesting that these are unique to the stem cell lines analyzed and may be pertinent to their specific behavior (Figure 5A). The median length of these mutations was approximately 28 kb and the total average genome coverage was 4.2 Mb per cell line. Further annotation using the ENTREZ gene database [14] mapped 181 genes to these unique CNVs. Out of these, 85 were found to be expressed in normal stem cell lines.

We matched this list of genes with the OMIM disease database [15] and found that 27 were previously implicated in a range of disorders encompassing different forms of cancer (CYLD, NOD2, SLC19A1, COL18A1), cardiovascular (ADRA1B, NEBL, NRG1, ZFPM2, UMOD) and psychiatric disorders (GABA3, DAOA, NRG1, KMO, CTNNA3, ZDHHC17, PPP2RB2, OPRD1). The primers for validation are given in Table S1.

We further detected 1269 LOH sites (Figure 5B). We matched these with the CNVs and further annotated them according to the Toronto Database of Variation. The number of annotated copy number neutral LOHs (or uniparental disomies (UPDs)) was 211, with a median length of 235 kb and coverage of 10 Mb per cell line. In total, 363 genes reported in the ENTREZ database [14] were located in the UPD regions. Out of these, 128 were found to be expressed in normal hESCs. Again, annotation to OMIM [15] revealed that several cancer-related genes were involved in these LOH regions (MLH1, ZMAT3, ADCY7, PIK3CA).

Even the smaller abnormalities involved potential oncogenes, as illustrated in Figure 4. Both openly malignant lines (2102Ep and CH-ES1) had multiple large deletions and insertions involving genes participating in the cell cycle, apoptosis, growth regulation and oncogenesis (Figure 4, Table S2, Table S3). These included, for example, MYC, BRCA2, p53, and others the numerous deleted and duplicated genes according to the pathways they reperesent are listed in detail in (Table S3). The results regarding genomic structure indicate the high instability of CH-ES1 and 2102Ep cells. Remarkably, several numerical abnormalities were observed in the HS401, HS293 and SIVF-02 hESC lines, as illustrated in Figure 5. Two genes were also verified for copy number variation on DNA level by quantitave real-time PCR, namely loss of *GRB10* in HS293 and loss of *MLLT1* in HS401 (Table 4)

**Table 4:** Primers used in real-time PCR.

Gene	Forward primer (5′-3′)	Reverse primer (5′-3′)	Size(bp)
GRB10-A	AGGTGCTGGGTAGCATGTTC	GGCTACAACACCCCACTGAC	130
GRB10-B	TGTAGGGCCTCCAGAATTGA	TTTCCATTGAGCATCAAAACAG	138
MLLT1-A	CGTCCAGGTGAGGTTAGAGC	CCAGAAGACCACCTTCTCCA	145
MLLT1-B	CTGACAGCGGCAGATGTTTA	GAGAAGAAAACGCGATCCTG	107
HEM3 *	TGCACGGCAGCTTAACGAT	AGGCAAGGCAGTCATCAAGG	202

It is noteworthy that alterations of several potential oncogenes (such as RAB6A) were also seen in these hESC lines, which induced only benign tumors in the mouse teratoma assay.

Validation of mRNA expression by quantitative real time PCR of a given gene with an increased copy number or a deletion, also showed corresponding modification of its mRNA level (Table 5). Totally 11 genes that were either duplicated or deleted in the different hESC lines were tested for their mRNA expression. The increased copy numbers and deletions were seen in the hESC line, H1, already at earliest available passage 22 as revealed by the validation assay. The only cell line that showed altered mRNA expression from what was expected was the highly malignant CH-ES1 with high level of genomic imbalances.

**Table 5:** Primers used in real-time quantitative PCR.

Gene	Forward primer (5′-3′)	Reverse primer (5′-3′)	Size(bp)
MLLT1	CAGCAAGCCTGAGAAGATCC	TTGAAGTGGCCAGTCTCCTC	150
PK1A	TCCTGGTTTCCTCTGCAAGT	CGTTGTGCATCTTCTTCACC	97
GRB10	GAAGCAGTACAACGCCCCTA	CTCTGCACAGAGCAACCTCA	94
FGFR1	TCCGTCAATGTTTCAGATGC	TTCCATCTTTTCTGGGGATG	144
EHMT1	AGAGGACAGCAGGACTTCCA	TTCCGAACTCAGGTCAGACTC	142
RAB6A	TTGCTGACAAGAGGCAAGTG	CAAAGCTGCTGCTACACGTC	134
MAP3K15	AGGGCGATAATGTTCTGGTG	TCTCAGGTGCCATGTACTGC	135
FAM49B	CATATTCTCCCACCCAGCAT	TGGCAGGATTTGTCATCTTG	107
JAK1	AACTGAAGTGGACCCCACAC	CACCTGCTCCCCTGTATTGT	130
FH	TCGATTTTTGGGTTCTGGTC	CCATGGTCATTGCTTCACAC	122
PTPN13	ACCTCCACCTGGTGTGCTAC	ATCTGAGCTGGTGCTTTGCT	133
GAPDH*	GCAGCCCTGGTGACCAG	GGGAAGGTGAAGGTCGGA	62

## Discussion

The hESC line CH-ES1 showed many characteristics typical of a teratocarcinoma-derived EC cell line. Spontaneous teratocarcinomas generally arise from primordial germ cells, typically in the testis, but also occasionally in the ovary or at non-gonad sites. Experimental teratocarcinomas may also be derived from ectopically transplanted embryos [12]. A single blastomere of a four-cell stage human embryo could therefore also form a teratocarcinoma. It is likely that the blastomere cell that gave rise to the CH-ES1 line had an abnormal genetic constitution, which is very common in human pre-implantation embryos [16], [17].

Human EC cells commonly have nearly triploid genomes and DNA content with gross chromosomal changes and a large number of variations [3]. It has been suggested that such tumor cells originate from a tetraploid derivative of primordial germ cells. These cells subsequently lose and rearrange their chromosomes to first generate a seminoma and then the more malignant and pluripotent EC cells, which stabilize at an approximately 3n DNA content [18], [19]. It is thus tempting to speculate that the blastomere that gave rise to CH-ES1 may have been tetraploid and that subsequent chromosomal loss resulted in an EC-like phenotype by a mechanism comparable to that by which EC cells arise.

Our results emphasize the importance of not only cytogenetic testing but also more detailed genetic testing of hESC lines by microarray methods before their clinical application in regenerative medicine. A large proportion of early human embryos are chromosomally abnormal, particularly those with poor morphology or developmental delays. The embryos donated for research are often of poor quality, but reported chromosomal abnormalities in hESC lines are not common, at least in early passages. For instance, all 30 hESC lines derived in our laboratory at Karolinska Institutet display karyotypically normal G-banding patterns [20]. It may be that genetically abnormal embryos do not form hESC lines as easily as normal ones. It is unlikely that the abnormalities in CH-ES1 would have been caused by the derivation process itself or by early culture, because we used identical conditions to those used to produce the 30 chromosomally normal hESC lines [20. Instead, derivation from a single blastomere may play a role, since Geens et al. [11], who succeeded in deriving hESC lines from embryos that were established for the study of early development, obtained a cytogenetically abnormal line.

There are several possible explanations for the malignancy of the CH-ES1 and the teratocarcinoma lines, including partial triploidy [21]. Many of the trisomies that have been identified in cancers and culture-adapted cells [4], [17], [18] were also seen in CH-ES1 cells, such as trisomy of chromosomes 1, 12, and X and a duplication of 17p13.2-qtel(3.674-tel). In addition, there were trisomies of chromosomes 9, 19, 20 and 21. In fact, only chromosome 14 was normal in the CGH assays of the CH-ES1 line. It is not difficult to understand why this particular cell line is particularly malignant and invasive. According to the CGH analysis, the changes observed in H1 (the oldest hESC line, which was at passage number 56 at the time of analysis) may have been there from the beginning. However, it is also possible that these changes arose during culture adaptation. We do not presently have CGH or SNP array data from earlier passages or from other laboratories.

Culture adaptation of hESCs and accumulation of chromosomal changes during long term culture occur as a result of the successive increase of selective growth advantages provided by certain abnormalities in the cells [4], [21], [22]. Furthermore, smaller changes than can be seen by G-banding have been described, and these may offer growth advantages similar to those that occur in cancer. Impaired imprinting and aberrations in mitochondrial DNA have been described [23], and impaired X-chromosome activation occurs during culture adaptation [24]. Culture adaptation has also been described in teratocarcinoma lines [3]. It is possible that least some of the aberrations in the studied lines are caused by culture adaptation. The aneuploidic increases in copy number of genes that may promote tumor formation, including ARHGAP26, GRB10, DDHD2, FGFR1, CTNNA3, PTPN1 and MLLT1 in the apparently stable and karyotypically normal lines HS293 and HS401, are cause for concern. Among the altered genes, ARHGAP26 and MLLT1 have been associated with leukemia-specific translocations, DDHD2, FGFR1 and PTPN1 have tumor-promoting potential in breast cancer, and CTNNA3 may promote tumor formation in urothelial cancer. Furthermore, a copy of the GRB10 gene, which acts as a growth inhibitor, was lost from HS293, supporting the idea of an acquired growth advantage. Such losses or gains of these potential growth- or cancer-promoting genes may increase the likelihood of malignant transformation with the accumulation of later mutations.

The SNP analysis was made using different passage levels to see what possible changes the lines displaying normal G-banding finding contain. In the quantitative PCR analysis of RNA expression, eleven genes were analysed for elevated or decreased expression according to the losses and gains in the different cell lineages. All the findings of the PCR validation were consistent with the SNP array results, but the malignant CH-ES-1 behaved differently.

Translocated genes may come under the influence of different promoters and enhancers disturbing and altering their gene expression. This is a possible explanation to decreased PTPN13 mRNA expression although the gene was shown to be duplicated, and an increased mRNA expression of FH although loss of a gene copy in the teratocarcinoma-like CH-ES1

Long term testing in immune-suppressed animals is neither an adequate nor a sufficient model to study cancer transformation of hESC lines. It will be difficult to exclude the possibility that cells carrying copy number alterations of growth-promoting or tumor suppressor genes have malignant potential by studying them in model organisms. In xeno-models, all tumorigenic cells are more likely to be rejected than in transplantation between individuals of the same species [4], [25]. Immunosuppression of the recipient makes the problem of possible tumor formation even more serious. The only way to avoid such risks is to use cells at the earliest possible passage number to decrease the likelihood of such changes.

According to the CGH and SNP array results, the profiles were consistent among all six cell lines studied. However, as expected, the higher resolution offered by the SNP arrays revealed 1275 additional changes smaller than 43 kb (the threshold of CGH resolution). In addition, SNP arrays can identify copy number neutral aberrations showing LOH, such as gene conversions and uniparental isodisomies. Altogether, we found 211 segments with a median length of 235 kb and coverage of 10 Mb per genome that showed LOH, and these have not been previously recorded as CNVs. In total, these regions contained 363 ENTREZ [14] genes, of which 128 were found to be expressed in normal hESCs. We conclude that the increased resolution offered by the SNP arrays is required for assessing potentially harmful alterations in hESC lines.

In conclusion, the first teratocarcinoma-like hESC line derived from a single blastomere showed many features typical of malignant cells, such as trisomies, duplications, deletions, and increased copy numbers of oncogenes, explaining its malignancy. In addition, benign and cytogenetically normal hESC lines also displayed many potentially tumorigenic genomic alterations, which may be due to the derivation method or to the prolonged culture conditions. Hence, at a minimum, SNP-profiling of the hESC lines before their use in regenerative medicine is important.

## Materials and Methods

The lines HS293 and HS401 were previously derived from fresh poor quality embryos that had been donated for research after informed consent in the Fertility Unit of the Karolinska University Hospital, Huddinge, Sweden, as described [26], [27]. They were derived using postnatal human skin fibroblasts as feeder cells and Knockout Serum Replacement (SR, Invitrogen)-containing medium. The Ethics Board of the Karolinska Institutet approved the derivation and research use of these lines. At the time of DNA extraction, HS293 was at passage number 47, and HS401 was at passage number 25. The lines have been karyotyped several times after derivation, and they were found repeatedly to be cytogenetically normal. After injection into SCID mice, they formed benign teratomas containing differentiated tissue components of the three germ layers.

The line CH-ES1 was derived under the same culture conditions as the lines produced at Karolinska Institutet using postnatal skin fibroblasts and SR-containing medium [5]. The derivation of this line was accomplished under the ethics permission and license of Swiss authorities. Surprisingly, the first karyotype performed at passage number three by G-banding showed many substantial chromosomal aberrations. Moreover, when CH-ES1 cells were injected into mice, they induced highly invasive tumors with clearly malignant cell composition [5]. At the time of DNA extraction, CH-ES1 was at passage number 19.

The clonal subline 2102Ep, an hEC line derived from a testicular teratocarcinoma, was maintained by one of us (PWA) in Sheffield as previously described [28]; DNA was extracted from the clone at passage number 40.

The hESC lines H1 from WiCell Research Institute (Madison, WI), SIVF02 (non-GMP line, a kind gift of Sydney IVF, Australia), CH-ES1 [5], HS293 and HS401 were maintained in DMEM/F-12 medium supplemented with 20% serum replacement, L-glutamine, non-essential amino acids, and 4 ng/ml human basic fibroblast growth factor. All hESC lines were cultured on irradiated human foreskin fibroblasts and passaged mechanically. The fibroblast feeders were cultured in DMEM (Invitrogen) supplemented with 10% fetal bovine serum and 1% penicillin/streptomycin (both from Invitrogen). Cells were mitotically inactivated by irradiation at 35 Gy before seeding on a gelatin-coated 6-well plate at 3.5×10^5^ cells/plate. The hESC culture medium was changed daily.

Prior to DNA extraction for SNP analysis, cells were cultured for at least four passages under feeder-free culture conditions on Matrigel Growth Factor Reduced (Becton Dickinson AG, Basel, Switzerland) coated 6-well plates with feeder-conditioned medium (CM). Matrigel was diluted 1∶30 with DMEM/F12 and 0.5 ml of the dilution was added to cover each well of a 6-well plate and allowed to gel for 1 h at 37°C. Plates were immediately used after the coating procedure. CM was prepared by incubating stem cell media overnight on irradiated feeder cells plated at the same density for hESC culture. CM was harvested after 24 h and supplemented with 20 ng/mL bFGF immediately before use with hESC cultures. This procedure was repeated for one week before discarding the feeder cells.

### Array CGH

DNA was extracted from cells using the QUIamp DNA extract kit (Qiagen Germantown, MD) following standard protocols. The same DNA samples were used for both SNP arrays and array-CGH.

Array-CGH was performed using the Agilent Human Genome CGH Microarray Kit 44B (Agilent Technologies, Santa Clara, California, USA). This platform is a high-resolution 60-mer oligonucleotide-based microarray that allows genome-wide surveys and molecular profiling of genomic aberrations with a resolution of ∼75 kb. Labeling and hybridization were performed following the protocols provided by Agilent. Briefly, 500 ng of purified DNA from a patient and a control (Promega Corporation, Madison, Wisconsin, USA) was double-digested with *RSAI* and *AluI* for two hours at 37°C. After twenty minutes at 65°C, each digested sample was labeled by the Agilent random primers labeling kit for two hours using Cy5-dUTP for the patient DNA and Cy3-dUTP for the control DNA. Labeled products were purified on columns and prepared according to the Agilent protocol. After probe denaturation and pre-annealing with 5 µl of Cot-1 DNA, hybridization was performed at 65°C with rotation for 40 hours. After two washing steps the arrays were analyzed with the Agilent scanner and Feature Extraction software (v9.1.3). A graphical overview was obtained using the CGH analytics software (v3.4.27).

The identification of aberrant chromosomal regions was performed manually using CGH-Analytics software (v3.4.27) (Agilent Technologies) according to the UCSC Genome Bioinformatics, (2010) [29] http://genome.ucsc.edu) and the Database of Genomic Variants 2010) [13] (http://projects.tcag.ca/variation/) on the Human March 2006 assembly.

Associations between genomic instability and Pathways, Gene Ontology and manually assembled gene lists were tested with R/bioconductor [30] and Webgestalt [31]. Losses and gains were considered separately, and enrichment was assessed with hypergeometric tests corrected for multiple testing using False Discovery Rate (FDR).

### SNP Arrays

The genotyping to detect both copy-number variations and loss of heterozygosity (LOH) without loss of chromosomal material was performed using the Affymetrix Genome-Wide Human SNP Array 6.0 (San Diego, CA). Labeling and hybridization were performed following the protocols provided by the manufacturer. The CRMA method [32] from the Aroma Affymetrix package [33] was used to asses total CNV.

As a CNV neutral reference group, we used data from a set of 20 arrays of nonmalignant blood cell DNA samples that had been previously hybridized in the same laboratory (JK).

To separate signal from noise, we considered only CNVs with intensities larger than one standard deviation of the raw copy number signal across all of the stem cell arrays. Moreover, we required CNVs to be tagged by at least four consecutive probes. Further testing by qPCR of CNVs close to the cut off confirmed the adequacy of this choice.

LOH was estimated using genotyping calls from Affymetrix proprietary software Genotyping Console (birdseed method) and a Hidden Markov Chain Method (HMCM) as implemented in the software dChip. LOH and CNVs were compared in order to determine Uniparental Disomy (UPD [34] or copy number neutral LOH.

### Verification of copy number variation with quantitative real-time PCR

Two selected variations in the hESC lines, the deletions of the GRB10 gene in HS293 and the MLLT1 gene in HS401, were verified by designing PCR amplicons within the deleted segments. A copy number neutral amplicon, HEM3, was used as a reference [35].

Two amplicons per gene were designed in the Primer Express v2.0 program (table 6). qRT-PCR analyses were performed in 20 µl volumes with 1 x Fast SYBR Green PCR Master Mix (Applied Biosystems), 10 ng genomic DNA and optimized primer concentrations: for HEM3 600 nmol/L, and for GRB10 and MLLT1 400 nmol/L. Each amplicon was quantified in triplicate using the Fast SYBR program (95°C for 20 s, followed by 40 cycles of 95°C for 3 s and 60°C for 30 s) on a 7500 Real-time PCR machine (Applied Biosystems). Relative copy number estimates were derived through ΔΔ Ct calculations for the copy number neutral amplicon, the HEM3 control gene. Three laboratory control DNA samples were used as standards for analyzing relative copy number.

**Table 6:** Summary of selected genes showing gains or losses in the different cell lineages and their corresponding mRNA expression.

Gene	Sample	Chr	Aberration	mRNA expression^#^
MLLT1	HS401	19	loss	2-fold decrease
PK1A	HS401	8	gain	2-fold increase
GRB10	HS293	7	loss	2-fold decrease
FGFR1	HS203	8	gain	2-fold increase
EHMT1	H1	9	loss	3 - 4-fold decrease*
RAB6A	H1	11	gain	1.3-2-fold increase*
MAP3K15	SIVF-02	X	loss	4-fold decrease
FAM49B	SIVF-02	8	gain	2-fold increase
JAK1	CH-ES1	1	loss	3-fold decrease
FH	CH-ES1	1	loss	1.5-fold increase
PTPN13	CH-ES1	4	gain	3-fold decrease

### RNA extraction and quantitative real time polymerase chain reaction

Total RNA was extracted from the different cell lineages using the Trizol reagent according to the manufacturer's instruction (Invitrogen). By the time of RNA extraction, HS401 was at passage 35, HS293 at passage 54, H1 was analysed from three different passage levels 22, 33 and 69, SIVF-02 at passage 45 and CH-ES 1 at passage 14. cDNA was synthesized from 1 µg of total RNA with the SuperScript II First-Strand synthesis system (Life Technologies). Quantitative RT-PCR measurements of individual cDNAs were performed in a final volume of 10 µl using SYBR green PCR master mix (Applied Biosystems) to measure duplex DNA formation with the 7500 Real-time PCR machine (Appplied Biosystems). Gene-specific primers were designed using the Primer3 software [35] with standard selection criteria in order to amplify approximately 90–150 bp long PCR fragments (table 5). Real-time PCR primers were used at a final concentration of 100 nM. Melting curve analysis and agarose gel electrophoresis was performed to monitor production of the appropriate PCR product. Each PCR reaction was performed in triplicates with negative controls. The results were normalized to endogenous *GAPDH* and *PSMB* mRNA levels.

### Bioinformatics

Putative phenotypically neutral CNV and UPD sites were purged by comparing the detected changes to the polymorphisms and aberrations from the database of Genomic Variants November 2008 Assembly (hg18) [13] (http://projects.tcag.ca/variation/). The remaining sites were annotated with the genes from the ENTREZ database (http://www.ncbi.nlm.nih.gov/sites/entrez) and the genes were further annotated to the Human disease database David, Bioinformatics Resources. NIH (2009) [36] (http://david.abcc.ncifcrf.gov/) and OMIM. NIH (2009) [15] (http://www.ncbi.nlm.nih.gov/OMIM).

### Expression microarrays

Microarray data on Affymetrix HGU133plus2 chips (San Diego, CA) that had been hybridized with normal stem cell lines (HS237 and HS181) in a previous experiment were used to evaluate gene activity. Presence calls from the Affymetrix MAS5 algorithm where used to establish whether a gene was expressed in normal stem cell lines. As the hybridization was performed in two technical replicates and genes could be interrogated by several probe sets, we designated a gene as expressed when it was present at least half of the time it was interrogated.

## Supporting Information

Table S1 Primers used in real-time quantitative PCR.
(0.03 MB DOC)

Table S2 Altered pathways in the studied lines.
(0.09 MB DOC)

Table S3 Deleted (DEL) and duplicated (DUPL) genes in H1, CH-ES1, and 2102 Ep cell lines related to several signaling pathways.
(0.07 MB DOC)
