# Supplementary material for: A Teratocarcinoma-Like Human Embryonic Stem Cell (hESC) Line and Four hESC Lines Reveal Potentially Oncogenic Genomic Changes
Source: PLoS One. 2010 Apr 23;5(4):e10263. doi: 10.1371/journal.pone.0010263 (PMC2859053; doi:10.1371/journal.pone.0010263)
Supplement: Table S1 — Primers used in real-time quantitative PCR. (0.03 MB DOC) [file pone.0010263.s001.doc]

Table S 1: Primers used in real-time quantitative PCR.

| **Gene** | **Forward primer (5´-3´)** | **Reverse primer (5´-3´)** | **Size (bp)** |
| --- | --- | --- | --- |
| **GRB10-A** | AGGTGCTGGGTAGCATGTTC | GGCTACAACACCCCACTGAC | 130 |
| **GRB10-B** | TGTAGGGCCTCCAGAATTGA | TTTCCATTGAGCATCAAAACAG | 138 |
| **MLLT1-A** | CGTCCAGGTGAGGTTAGAGC | CCAGAAGACCACCTTCTCCA | 145 |
| **MLLT1-B** | CTGACAGCGGCAGATGTTTA | GAGAAGAAAACGCGATCCTG | 107 |
| **HEM3 *** | TGCACGGCAGCTTAACGAT | AGGCAAGGCAGTCATCAAGG | 202 |
