# Supplementary material for: A Teratocarcinoma-Like Human Embryonic Stem Cell (hESC) Line and Four hESC Lines Reveal Potentially Oncogenic Genomic Changes
Source: PLoS One. 2010 Apr 23;5(4):e10263. doi: 10.1371/journal.pone.0010263 (PMC2859053; doi:10.1371/journal.pone.0010263)
Supplement: Table S2 — Altered pathways in the studied lines. (0.09 MB DOC) [file pone.0010263.s002.doc]

| Table S 2 | | | | | | |
| --- | --- | --- | --- | --- | --- | --- |
| **Sample** | **Chr** | **Type** | **Express** | **KEGG_PATHWAY** | **DB_DISEASE_CLASS** | **OMIM_DISEASE** |
| SIVF-02 | 1 | UPD | Expr |  |  | Charcot-Marie-Tooth disease |
| SIVF-02 | 1 | UPD | Expr | Lysine degradation | METABOLIC | Ehlers-Danlos syndrome, type VI, Nevo syndrome, |
| SIVF-02 | 1 | UPD | Expr |  | CANCER |  |
| SIVF-02 | 1 | UPD | Expr | interactions in vesicular transport | METABOLIC |  |
| SIVF-02 | 2 | UPD | OExpr |  | IMMUNE |  |
| SIVF-02 | 3 | UPD | Expr | Endometrial cancer | AGING, CANCER | Cafe-au-lait spots with glioma or leukemia, Colorectal cancer |
| SIVF-02 | 10 | UPD | Expr | Sulfur metabolism | METABOLIC | SEMD, Pakistani type, |
| SIVF-02 | 11 | UPD | Expr |  | NEUROLOGICAL |  |
| HS401 | 2 | UPD | OExpr | Natural killer cell mediated cytotoxicity | METABOLIC | Fibromatosis, gingival, Noonan syndrome 4, |
| HS401 | 5 | UPD | Expr | Cytokine-cytokine receptor interaction | DEVELOPMENTAL |  |
| HS401 | X | LOSS | Expr | ABC transporters - General |  | Anemia, sideroblastic, with ataxia, |
| HS293 | 3 | UPD | Expr | Apoptosis | CANCER | Breast cancer, Colorectal cancer, Ovarian cancer, |
| HS293 | 3 | UPD | Expr | p53 signaling pathway |  |  |
| HS293 | 4 | UPD | Expr | Ubiquitin mediated proteolysis |  |  |
| HS293 | 6 | UPD | OExpr |  |  | Leber congenital amaurosis V, |
| HS293 | 6 | UPD | Expr |  | METABOLIC |  |
| HS293 | 7 | LOSS | Expr |  | REPRODUCTION |  |
| HS293 | 8 | GAIN | OExpr | Melanoma | DEVELOPMENTAL | Jackson-Weiss syndrome, Kallmann syndrome 2 |
| HS293 | 9 | UPD | Expr | Long-term depression |  | Bleeding diathesis due to GNAQ deficiency, |
| H1 | 4 | UPD | Expr |  |  | Pulmonary fibrosis, idiopathic, |
| H1 | 7 | UPD | OExpr |  | CARDIOVASCULAR |  |
| H1 | 11 | UPD | Expr |  |  | Fanconi anemia, complementation group F, |
| H1 | 16 | UPD | OExpr |  | NEUROLOGICAL | Charcot-Marie-Tooth disease, type 1C, |
| H1 | 18 | UPD | Expr | Cytokine-cytokine receptor interaction | DEVELOPMENTAL | Osteolysis, familial expansile, Paget disease of bone, |
| H1 | X | LOSS | Expr |  | REPRODUCTION |  |
| ChES1 | 1 | LOSS | Expr | Renal cell carcinoma | CANCER | Fumarase deficiency, Leiomyomatosis and renal cell cancer |
| ChES1 | 3 | LOSS | Expr |  |  | Bardet-Biedl syndrome 1 |
| ChES1 | 4 | GAIN | Expr |  | IMMUNE |  |
| ChES1 | 5 | UPD | Expr | hsa19Oxidative phosphorylation |  | Complex I, mitochondrial respiratory chain |
| ChES1 | 6 | GAIN | Expr |  | IMMUNE | Leukemia, acute nonlymphocytic, |
| ChES1 | 8 | UPD | Expr |  | NEUROLOGICAL | Charcot-Marie-Tooth disease, type 4D, |
| ChES1 | 10 | GAIN | Expr | Adipocytokine signaling pathway |  |  |
| ChES1 | 12 | UPD | Expr |  |  | Bardet-Biedel syndrome 1, |
| ChES1 | 13 | GAIN | Expr | Streptomycin biosynthesis |  |  |
| ChES1 | 14 | GAIN | Expr | ABC transporters - General |  |  |
| ChES1 | 14 | GAIN | Expr |  |  | Microphthalmia, isolated 2, Microphthalmia |
| ChES1 | 16 | UPD | Expr | Melanogenesis |  |  |
| ChES1 | 20 | UPD | Expr |  | CARDIOVASCULAR |  |
| ChES1 | 20 | UPD | Expr |  |  |  |
| ChES1 | 21 | UPD | OExpr | Alzheimer's disease | NEUROLOGICAL, | Alzheimer disease |
| ChES1 | X | GAIN | Expr | Ubiquitin mediated proteolysis |  | Opitz G syndrome, type I, |
| 2102EP | 1 | GAIN | Expr | Nitrogen metabolism | CANCER | Cystathioninuria, Homocysteine, total plasma, elevated, |
| 2102EP | 3 | UPD | Expr | Ribosome |  |  |
| 2102EP | 5 | GAIN | Expr | Cell Communication |  | Leukodystrophy, adult-onset, autosomal dominant, |
| 2102EP | 5 | GAIN | Expr |  |  | Epilepsy, pyridoxine-dependent, |
| 2102EP | 5 | GAIN | Expr | Ubiquitin mediated proteolysis |  |  |
| 2102EP | 5 | GAIN | Expr | Cell cycle | CANCER |  |
| 2102EP | 6 | UPD | Expr |  | NEUROLOGICAL | Myoclonic epilepsy, juvenile, 1, |
| 2102EP | 6 | UPD | Expr | Cell cycle |  |  |
| 2102EP | 7 | UPD | Expr | Proteasome |  |  |
| 2102EP | 7 | UPD | Expr | Gluconeogenesis |  | Hemolytic anemia due to bisphosphoglycerate mutase deficiency, |
| 2102EP | 10 | UPD | Expr |  | NEUROLOGICAL |  |
| 2102EP | 10 | UPD | Expr |  |  | Goldberg-Shprintzen megacolon syndrome, |
| 2102EP | 12 | GAIN | Expr |  |  | Bardet-Biedel syndrome 1, |
| 2102EP | 13 | GAIN | Expr |  | NORMALVARIATION |  |
| 2102EP | 16 | GAIN | Expr | Melanogenesis |  |  |
| 2102EP | 17 | GAIN | Expr | p53 signaling pathway |  | Breast cancer, |
| HS401 | 19 | LOSS | Expr |  |  | Leukemia |
