# Supplementary material for: A Teratocarcinoma-Like Human Embryonic Stem Cell (hESC) Line and Four hESC Lines Reveal Potentially Oncogenic Genomic Changes
Source: PLoS One. 2010 Apr 23;5(4):e10263. doi: 10.1371/journal.pone.0010263 (PMC2859053; doi:10.1371/journal.pone.0010263)
Supplement: Table S3 — Deleted (DEL) and duplicated (DUPL) genes in H1, CH-ES1, and 2102 Ep cell lines related to several signaling pathways. (0.07 MB DOC) [file pone.0010263.s003.doc]

**Table S 3.**

| **Pathway** | **No.**  **genes** | **Deleted/Duplicated Genes in Pathway** |
| --- | --- | --- |
| **Wnt Signaling** | | |
| 2102 Ep_DEL | 2 | FOSL1, RPL13A |
| 2102 Ep_DUPL | 38 | AES, AXIN1, BCL9, CCND1, CCND2, CSNK1A1, CSNK1D, CSNK2A1, CTNNB1, CTNNBIP1,  DVL1, DVL2, FBXW11, FGF4, FOXN1, FZD2, JUN, LRP6, NKD1, NLK, PORCN, PPP2CA,  SFRP4, SLC9A3R1, TCF7, TLE2, WNT1, WNT2B, WNT3, WNT4, WNT5A, WNT5B,  WNT7A, WNT8A, WNT9A, HPRT1, GAPDH, ACTB |
| CH-ES1_DEL | 15 | AXIN1, CSNK1G1, CTBP1, CXXC4, EP300, FZD3, KREMEN1, NKD1, PITX2, PORCN,  PPP2R1A, PYGO1, SFRP4, B2M, RPL13A |
| CH-ES1_DUPL | 5 | FBXW11, RHOU, WNT2B, WNT3A, WNT9A |
| H1_DEL | 1 | RPL13A |
| H1_DUPL | 4 | BCL9, JUN, WNT2B, WNT4 |
| **Stem Cell Markers** | | |
| 2102 Ep_DEL | 3 | BMP1, NCAM1, RPL13A |
| 2102 Ep_DUPL | 38 | ADAR, ALDH2, AXIN1, BGLAP, BMP2, CCND1, CCND2, CD4, CDC42, CDH1, COL1A1,  COL2A1, CTNNA1, DHH, DTX1, DTX2, DVL1, FGF1, FGF3, FGF4, FOXA2, GDF3, GJB1,  HSPA9, IGF1, JAG1, KRT15, MYST1, MYST2, NOTCH2, OPRS1, PARD6A, PPARG, S100B,  WNT1, HPRT1, GAPDH, ACTB |
| CH-ES1_DEL | 20 | ABCG2, AXIN1, BMP1, BMP3, CCNA2, CD8A, CD8B, CDH1, EP300, FGF2, GJB2, PDX1,  MSX1, NEUROG2, PARD6A, PPARG, RB1, TUBB3, B2M, RPL13A |
| CH-ES1_DUPL | 3 | ADAR, BGLAP, PPARD |
| H1_DEL | 1 | RPL13A |
| H1_DUPL | 4 | ADAR, BGLAP, CDC42, NOTCH2 |
| **Cancer Pathway** | | |
| 2102 Ep_DEL | 5 | ANGPT2, ATM, MMP1, TNFRSF10B, RPL13A |
| 2102 Ep_DUPL | 35 | APAF1, BAX, BCL2L1, BRCA1, CDC25A, CDK2, CDK4, CDKN2A, COL18A1, E2F1, ERBB2,  IFNA1, IFNB1, IGF1, ITGA3,TGB3, JUN, MDM2, MMP2, MMP9, NME1, NME1-NME2,  NME4, RAF1, S100A4, TEK, TIMP1, TNFRSF1A, TNFRSF25, TP53, TWIST1, EPDR1,  HPRT1, GAPDH, ACTB |
| CH-ES1_DEL | 19 | ANGPT2, BCL2, CDC25A, CHEK2, IL8, MMP2, NFKB1, NME4, PDGFB, RAF1, RB1,  SERPINB5, TGFB1, TIMP1, TIMP3, TNFRSF10B, EPDR1,B2M, RPL13A |
| CH-ES1_DUPL | 2 | CDKN1A, MMP9, c-Myc |
| H1_DEL | 2 | BCL2L1, RPL13A |
| H1_DUPL | 2 | JUN, S100A4 |
| **Apoptosis** | | |
| 2102 Ep_DEL | 5 | BIRC2, BIRC3, TNFRSF10A, TNFRSF10B, RPL13A |
| 2102 Ep_DUPL | 35 | APAF1, BAG1, BAX, BCL10, BCL2L1, BFAR, XIAP, BIRC6, BNIP1, BRAF, NOD1,  CASP14, CASP9, CD40, CD40LG, CRADD, DFFA, GADD45A, HRK, LTBR, MCL1,  NOL3, PYCARD, TNFRSF1A, TNFRSF25, CD27, TNFRSF9, CD70, TP53, TP73,  TRADD, TRAF4, HPRT1, GAPDH, ACTB |
| CH-ES1_DEL | 20 | BCL2, BCL2L10, BFAR, BID, BIK, BIRC6, BIRC8, BNIP3L, NOD1, CARD8,  CASP3, CASP6, CIDEA, NOL3, PYCARD, NFRSF10A, TNFRSF10B, TRADD,  B2M, RPL13A |
| CH-ES1_DUPL | 7 | BCL10, BCLAF1, BNIP1, BRAF, CASP2, CD40, TP53BP2 |
| H1_DEL | 2 | BCL2L1, RPL13A |
| H1_DUPL | 4 | BCL10, FASLG, GADD45A, MCL1 |
| **Cell Cycle** | | |
| 2102 Ep_DEL | 6 | ATM, BRCA2, CDC16, MRE11A, TFDP1, RPL13A |
| 2102 Ep_DUPL | 35 | DIRAS3, BAX, BIRC5, BRCA1, CCND1, CCND2, CCNF, CCNT1, CDC20, CDC34,  CDK2, CDK4, CDK5R1, CDK5RAP1, DKN1B, CDKN2A, CDKN2B, CKS1B, DDX11,  DNM2, E2F4, GADD45A, HUS1, KNTC1, KPNA2, MAD2L2, PCNA, RBL1,  RBL2, RPA3, TP53, UBA1, HPRT1, GAPDH, ACTB |
| CH-ES1_DEL | 22 | ANAPC4, BCL2, BRCA2, CCNB2, CCNF, CDC16, CDC34, CDK8, CHEK2, E2F4,  GTSE1, HERC5, HUS1, MCM5, RAD51, RB1, RBL2, SERTAD1, TFDP1, UBA1,  B2M, RPL13A |
| CH-ES1_DUPL | 2 | CDKN1A, CKS1B |
| H1_DEL | 1 | RPL13A |
| H1_DUPL | 4 | DIRAS3, CDC20, CKS1B, GADD45A |
| **Growth Factors** | | |
| 2102 Ep_DEL | 4 | BMP1,FGF14, FGF17, RPL13A |
| 2102 Ep_DUPL | 37 | AMH, BMP2, BMP7, BMP8B, CSF1, CSF2, CSF3, CSPG5, FGF1, FGF11, FGF13,  FGF19, FGF22, FGF23, FGF6, FIGF, GDF11, HBEGF, IGF1, IL12B, IL3, IL4,  JAG1, LEFTY1, LEFTY2, NDP, NGF, NRG2, NRTN, NTF3, PSPN, SLCO1A2,  TDGF1, THPO, HPRT1, GAPDH, ACTB |
| CH-ES1_DEL | 23 | BMP1, BMP3, CSPG5, CXCL1, FGF14, FGF17, FGF2, FGF22, FGF7, FGF9, FIGF,  IL2, LIF, LTBP4, NDP, NRTN, OSGIN1, SPP1, TDGF1, TGFB1, TNNT1, B2M, RPL13A |
| CH-ES1_DUPL | 4 | CSF1, LEFTY1, LEFTY2, PTN |
| H1_DEL | 1 | RPL13A |
| H1_DUPL | 4 | BMP8B, CSF1, IL10, NGF |
| **Notch Signaling** | | |
| 2102 Ep_DEL | 6 | CDC16, FOSL1, HR, MMP7, ZIC2, RPL13A |
| 2102 Ep_DUPL | 36 | ADAM17, AES, AXIN1, CCND1, CTNNB1, DTX1, ERBB2, FIGF, FZD2, GBP2, GLI1,  HDAC1, HEYL, HOXB4, IFNG, IL17B, AG1, KRT1, LFNG, MAP2K7, MYCL1,  COR2, NOTCH2, NOTCH3, PAX5, PDPK1, POFUT1, PPARG, RFNG, RUNX1,  SH2D1A, STIL, STAT6. HPRT1, GAPDH, ACTB |
| CH-ES1_DEL | 15 | ADAM10, ADAM17, AXIN1, CDC16, EP300, FIGF, FZD3, HR, MFNG, NFKB1,  PDPK1, PPARG, ZIC2, B2M, RPL13A |
| CH-ES1_DUPL | 3 | CDKN1A, GBP2, PSEN2 |
| H1_DEL | 2 | POFUT1, RPL13A |
| H1_DUPL | 8 | GBP2, HDAC1, HEYL, LOR, MYCL1, NOTCH2, NOTCH2NL, STIL |
| **TGFβ pathway** | | |
| 2102 Ep_DEL | 3 | BMP1, TSC22D1, RPL13A |
| 2102 Ep_DUPL | 39 | ACVRL1, AMH, AMHR2, BGLAP, BMP2, BMP7, BMPER, CDC25A, CDKN2B,  CER1, CHRD, COL1A1, CST3, FKBP1B,GDF3, GDF5, GDF7, HIPK2, ID1, ID2,  IGF1, IGFBP3,IL6, ITGB7, JUN, JUNB, LEFTY1, LTBP1, NBL1, NOG, NR0B1,  RUNX1, SMAD5, TGFB1I1, TGFBI, TGFBR3, HPRT1, APDH, ACTB |
| CH-ES1_DEL | 17 | BMP1, BMP3, CDC25A, FKBP1B, LTBP4, NOG, NR0B1, PDGFB, SMAD2, S  MAD4, TGFB1, TGFB1I1, TSC22D1, TGFBR2, TGIF1, B2M, RPL13A |
| CH-ES1_DEL | 4 | BGLAP, CDKN1A, HIPK2, LEFTY1 |
| CH-ES1_DUPL | 2 | ID1, RPL13A |
| H1_DUPL | 3 | BGLAP, JUN, TGFBR3 |
|  |  |  |
